# Cold Feet

**Published:** 1881-04

**Authors:** 


### COLD FEET.

Says the London *Lancet:* It is, as we have often labored to show, a mistake to suppose there is any warmth in clothes. Animal heat is the direct result of changes going on within the body itself. Nutrition by food and the discharges of energy by exercise are the efficient causes of heat. Clothes "seem" good and warm because they prevent the cold air and objects with a capacity for heat which surround the body from attracting the heat generated within its organism. The clothing is simply an insulator. It follows that it should be light in weight, and above all things, that it should permit the free and full circulation of blood through every part of the system—to the end of every finger and toe—and that the muscular apparatus of the extremities should be in perfect working order. If we will wear foot coverings, whether boots or stockings, which compress the feet and render the separate action of each toe impossible, it is simply absurd to expect to be warm-footed. Heat is the complement of work and nutrition, and if a part of the organism is so bound that it cannot work, and its supply of food is limited, it must be cold. The resort to stouter and heavier clothing is simply ridiculous. Generally it is the stockings that compress the feet. The garter acts as a ligature, and diminishes the blood supply, while the stocking itself acts as a bandage, and impedes the circulation through the extremities.

### HE TOOK THE HINT.

Young Mr. Latehours was sitting on the porch the other night watching a seventeen-year-old girl trying to keep awake long enough to see the morning star rise. They talked astronomy.

"I wish I was a star," he said, smiling at his own poetic fancy.

"I would rather you were a comet," she said, dreamily.

His heart beat tumultuously.

"And why?" he asked, tenderly.

"Oh, she said, with a brooding earnestness that fell upon his soul like a bare foot on a cold oilcloth, "because then you would only come around once every 1,500 years."

He didn't say anything until he was half way to the front gate, when he turned round and shook his fist at the house, and muttered between his teeth that "it would be longer than that before he came around again." But by that time the poor girl was in bed and sound asleep.—*Burlington Hawkeye.*

